# Gaps in guideline-recommended anticoagulation in patients with atrial fibrillation and elevated thromboembolic risk within an integrated healthcare delivery system

**DOI:** 10.1186/s12872-023-03607-y

**Published:** 2023-11-21

**Authors:** Sushmita Malik, Shanshan Gustafson, Huai-En R. Chang, Yonas Tamrat, Alan S. Go, Natalia Berry

**Affiliations:** 1 Department of Internal Medicine, Kaiser Permanente Mid-Atlantic States, Rockville, Maryland USA; 2 Division of Research, Kaiser Permanente Northern California, Oakland, California USA; 3 Department of Cardiology, Kaiser Permanente Mid-Atlantic States, Rockville, Maryland USA

**Keywords:** Anticoagulation rates, Elevated thromboembolic risk, Atrial fibrillation

## Abstract

**Background:**

Atrial Fibrillation (AF) is the leading cause of stroke, which can be reduced by 70% with appropriate oral anticoagulation (OAC) therapy. Nationally, appropriate anticoagulation rates for patients with AF with elevated thromboembolic risk are as low as 50% even across the highest stroke risk cohorts. This study aims to evaluate the variability of appropriate anticoagulation rates among patients by sex, ethnicity, and socioeconomic status within the Kaiser Permanente Mid-Atlantic States (KPMAS).

**Methods:**

This retrospective study investigated 9513 patients in KPMAS’s AF registry with CHADS_2_ score ≥ 2 over a 6-month period in 2021.

**Results:**

Appropriately anticoagulated patients had higher rates of diabetes, prior stroke, and congestive heart failure than patients who were not appropriately anticoagulated. There were no significant differences in anticoagulation rates between males and females (71.8% vs. 71.6%%, [OR] 1.01; 95% CI, 0.93-1.11; *P* = .76) nor by SES-SVI quartiles. There was a statistically significant difference between Black and White patients (70.8% vs. 73.1%, *P* = .03) and Asian and White patients (68.3% vs. 71.6%, *P* = .005). After adjusting for CHADS_2_, this difference persisted for Black and White participants with CHADS_2_ scores of ≤3 (62.6% vs. 70.6%, *P* < .001) and for Asian and White participants with CHADS_2_ scores > 5 (68.0% vs. 79.3%, *P* < .001).

**Conclusions:**

Black and Asian patients may have differing rates of appropriate anticoagulation when compared with White patients. Characterizing such disparities is the first step towards addressing treatment gaps in AF.

**Supplementary Information:**

The online version contains supplementary material available at 10.1186/s12872-023-03607-y.

## Background

Atrial fibrillation (AF) is the most common clinicallyf meaningful arrhythmia in adults, estimated to affect up to 6 million patients in the United States [1, 2]. AF is one of the leading causes of ischemic stroke, which can be reduced by up to 70% with the use of oral anticoagulation (OAC) therapy [1].

Nationally, guideline-recommended OAC for adults with AF who have an elevated predicted ischemic stroke risk using the CHA_2_DS_2_VASc score is reported at 50% even within the highest stroke risk cohorts [3]. A higher CHA_2_DS_2_VASc score is associated with a higher risk of ischemic stroke in patients with AF, but this risk score has limitations in terms of its accuracy to predict annual absolute risk of ischemic stroke [1, 4], with the recent ATRIA (Anticoagulation and Risk Factors in Atrial Fibrillation) risk score having been validated as more accurate predictor of ischemic stroke [5–7]. Given that stroke is the leading cause of disability in the US, and contributes to morbidity, premature death, and high costs, understanding the gaps in guideline-recommended OAC in understudied patient groups is an important first step towards decreasing the risk of stroke in the growing population with AF nationally [1].

There are conflicting data on guideline-recommended OAC use by sex, with some prior studies suggesting that women with AF are less likely than men to use OAC [8–10], while others reporting similar rates between males and females [11]. Although AF incidence appears to be lower in Black, Hispanic, and Asian patients compared to White patients [2], limited studies suggest that Black and Hispanic patients [12, 13] as well as Native American and Alaskan Native patients may have lower rates of OAC for stroke prevention in AF [14]. In addition, lower socioeconomic status has been reported to be associated with a lower likelihood of treatment with direct oral anticoagulants (DOACs) for AF [15]. However, existing studies focusing on these sociodemographic and other patient subgroups with AF have important limitations, including modest sample sizes, limited diversity, or samples from an earlier treatment era.

To address these knowledge gaps, we examined variability in OAC rates across sex, ethnicity, and socioeconomic status in a contemporary, diverse cohort of adults with AF receiving care within a large integrated health care delivery system.

## Methods

### Source population

This source population was based in Kaiser Permanente Mid-Atlantic States (KPMAS), a large, integrated health care delivery system providing comprehensive care for approximately 800,000 patients throughout the District of Columbia, Maryland, and Northern Virginia. The KPMAS membership is highly diverse across age, sex, race, ethnicity, and socioeconomic status. Information on clinical care on all members is systematically captured through an Epic®-based electronic health record (EHR) system.

This study was approved by the KPMAS institutional review board. A waiver of informed consent was obtained given the nature of the study.

### Study sample

We performed a retrospective cohort study of adult (age ≥ 18 years) patients within the KPMAS AF Registry considered at moderate or higher stroke risk based on having a CHADS_2_ score of 2 or greater between March 1, 2021 through September 31, 2021. We were unable to calculate either a CHA_2_DS_2_VASc score or an ATRIA stroke risk score based on available data in the registry.

### Receipt of anticoagulation and potential reasons for not receiving anticoagulation

We ascertained receipt of OAC (i.e., warfarin or a DOAC) based on information on the patient’s active medication list in the EHR. Systematic information on absolute or relative contraindications were unavailable on all AF Registry members, so we performed manual EHR review of a randomly selected subset of patients who did not receive guideline-recommended OAC to determine potential reasons for not receiving OAC.

### Patient characteristics

Patient age, sex and self-reported race and ethnicity information was ascertained from EHR data. Socioeconomic status was classified from U.S. Census tract data using social vulnerability index (SVI-SES) which ranks each census tract on each of 14 factors that are grouped within four themes (i.e., economic and educational attainment status, household composition and disability, minority status and language, and housing and transportation) [16]. In addition, information on selected cardiovascular risk factors were obtained from EHR data sources, including the presence of hypertension, diabetes mellitus, prior ischemic stroke, chronic heart failure, coronary heart disease, and peripheral artery disease (see Supplemental Appendix for definitions).

### Statistical approach

All analyses were conducted using SAS statistical software, version 9.4 (Cary, N.C.). We compared baseline characteristics between patients with AF who did or did not receive guideline-recommended OAC using Student’s t test for continuous variables, and rates of guideline-recommended OAC were compared across subsets of sex, race/ethnicity, and SVI-SES quartile using chi-squared tests.

## Results

### Baseline characteristics and rates of guideline-recommended oral anticoagulation

A total of 9513 eligible patients with AF considered at increased stroke risk were identified between March 1, 2021-September 30, 2021.

Overall, 6823 (71.7%) of patients received guideline-recommended OAC. Among these patients, 33.8% received warfarin, 53.2% received dabigatran, 9.3% received apixaban, and 4.7% received rivaroxaban. The study cohort had mean (SD) age of 74.5 (10.8) years, 41.0% were women, and there was 49.1% White patients, 35.5% Black patients, 8.3% Asian or Pacific Islander patients, 4.8% Hispanic patients, and 2.3% patients of other racial/ethnic groups. Those receiving guideline-recommended OAC were more likely to have diabetes, prior stroke, and chronic heart failure (Table 1).

**Table 1:** Baseline characteristics of adults with AF at increased stroke risk based on CHADS_2_ score ≥ 2

Characteristic	Anticoagulated (%) (*N* = 6823)	Not Anticoagulated (%) (*N* = 2690)	*P* value
Mean (SD) age, yr	74.7 (9.8)	74.3 (12.2)	0.76
Men	4031 (59.1)	1580 (58.7)	0.77
Hypertension	5732 (84.0)	2251 (83.7)	0.69
Diabetes mellitus	2837 (41.6)	1035 (38.5)	0.006
Prior ischemic stroke	1193 (17.5)	302 (11.2)	< 0.001
CHF	2740 (40.2)	803 (29.9)	< 0.001
Prior CAD or PAD	2057 (30.1)	780 (29.0)	0.28

### Guideline-recommended oral anticoagulation in patient subgroups

Rates of guideline-recommended OAC anticoagulation were evaluated across strata of CHADS_2_ scores. Unadjusted rates of OAC use were higher with higher predicted stroke risk but plateaued for CHADS_2_ stroke risk score of 4 and higher: 68.2% for CHADS_2_ of ≤3 (*N* = 2139), 76.3% for CHADS_2_ of 4 (*N* = 2904), 78.4% for CHADS_2_ of 5 (*N* = 2007), and 77.6% for CHADS_2_ of ≥6 (*N* = 1796).

**Fig. 1:**
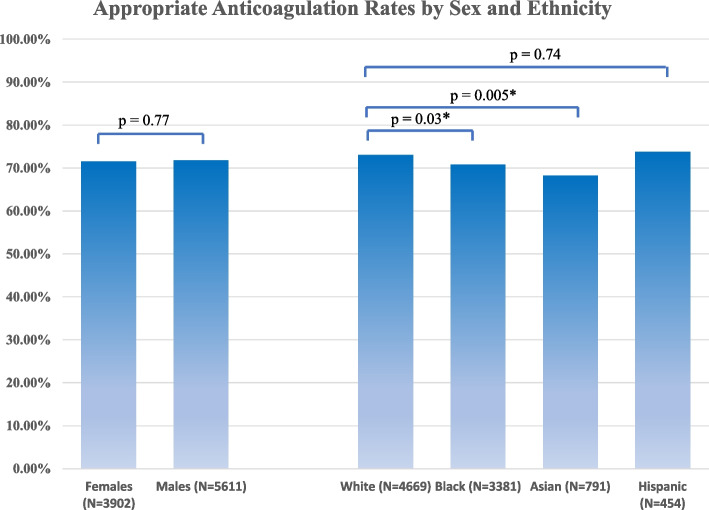
Rate of guideline-recommended oral anticoagulation by sex and ethnicity

There was no significant difference in the crude rate of guideline-recommended OAC between males and females (71.8% vs. 71.6%, *p* = 0.76) (Fig. 1, nor in rates across quartiles of SVI-SES (Table 2).

**Table 2:** Rate of guideline-recommended oral anticoagulation use by social vulnerability index socioeconomic status (SVI-SES) quartile

SVI SES Percentile	Anticoagulated (%)	*P* value
< 25%	3072 (72.0%)	0.53
25-49%	1792 (72.4%)	
50-74%	1318 (71.1%)	
> = 75%	449 (69.8%)	

Compared with White patients, we observed modestly lower unadjusted rates of guideline-recommended OAC in Black patients (73.1% vs. 70.8%, *p* = 0.03) and Asian patients (73.1% vs. 68.3%, *p* = 0.005) **(**Fig. 1**).** After adjustment by CHADS_2_ stroke risk score, this difference persisted for Black and White patients with CHADS_2_ scores of ≤3 (62.6% vs. 70.6%, *P* < .001) and for Asian or Pacific Islander and White patients with CHADS_2_ scores > 5 (68.0% vs. 79.3%, *P* < .001), (Table 3).

**Table 3:** Receipt of guideline-recommended oral anticoagulation by ethnicity and CHADS2 risk score

CHADS_2_ score	Anticoagulated (%)	*P* value (vs. White)
≤3		
White	855 (70.6%)	
Black	456 (62.6%)	< 0.001
Asian	148 (74.4%)	0.28
4		
White	1225 (76.6%)	
Black	803 (76.3%)	0.89
Asian	189 (74.4%)	0.66
5		
White	814 (80.1%)	
Black	630 (78.9%)	0.51
Asian	130 (67.7%)	< 0.001
≥6		
White	650 (78.2%)	
Black	637 (78.6%)	0.83
Asian	106 (68.4%)	0.008

### Reasons for not receiving guideline-recommended oral anticoagulation

**Fig. 2:**
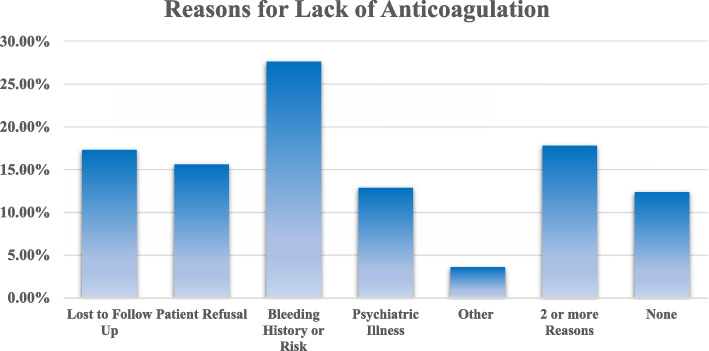
Reasons for not receiving OAC in patients with AF at increased stroke risk

Manual EHR review was performed for 225 randomly selected patients with AF who were not receiving guideline-recommended OAC (Fig. 2). A substantial portion (27.6%) of the patients reviewed had a documented bleeding history or a high predicted risk of bleeding. In 15.6% of patients, there was documented refusal to take OAC. An additional 17.3% of patients were lost to follow-up, 12.9% had significant psychiatric illness, and 3.6% had other reasons (e.g., history of LAA closure, or an occupation which precluded the ability to be anticoagulated). A total of 17.8% of patients had more than one reason for not receiving OAC. Overall, only 12.4% of the patients had no documented reason for not receiving OAC.

## Discussion

In a contemporary, diverse cohort of 9513 patients with AF considered at increased stroke risk receiving care within an integrated health care delivery system, 72% were receiving guideline-recommended use of OAC. Furthermore, manual review of medical records in a random sample of patients not receiving OAC suggests only a small proportion of such patients were eligible or were willing to be anticoagulated. This study highlights how an integrated health care system, such as Kaiser Permanente, can positively impact the treatment of chronic conditions like Atrial Fibrillation.

Rates of anticoagulation in our population are higher than reported in the National Cardiovascular Data Registry-PINNACLE Registry, 57% (NCDR-PINNACLE) [17], and similar to the smaller Outcomes Registry for Better Informed Treatment of Atrial Fibrillation, 76.2% (ORBIT-AF) [11]. The NCDR-PINNACLE noticed a plateau effect of OAC penetration among eligible AF patients considered at higher stroke risk (i.e., CHADS_2_ scores ≥2) [3]. Our data demonstrated numerically higher prescription rates of OAC therapy across higher CHADS_2_ scores, with a plateau effect evident at a CHADS_2_ score of 4. Given that the patients with elevated CHADS_2_ scores are at the highest predicted risk for thromboembolic events, appropriate treatment with OAC has a greater absolute benefit in reducing stroke burden among these patients. These findings may be attributable to the efficiencies of an integrated healthcare delivery system, which allows not only for rapid multispecialty communication, but access to care and reduced reimbursement barriers.

Dabigatran is currently the preferred OAC choice for stroke prevention in AF at KPMAS. In contrast, the OAC therapy in the NCDR PINNACLE and ORBIT-AF was predominantly warfarin, while the subsequent ORBIT-AF II registry established in 2014 reported OAC rates of 22% warfarin and 41% rivaroxaban, reflecting a shift towards the convenience and possibly lower risk profile of the newer DOAC classes. Interestingly, our data reported 33.8% warfarin use, possibly reflecting the burden of comorbidities in our population (CKD, advanced age) which would preclude the safe use of dabigatran. Despite the higher use of warfarin, appropriate anticoagulation rates remained relatively high as reported in our study.

### Sex

Female sex is an independent predictor of ischemic stroke risk in patients with AF off OAC [9, 11, 18], and results of reported appropriate OAC use by sex are mixed. Previous research including a retrospective study of Medicare beneficiaries [8] and a recent study found that females have lower initiation rate of any OACs, including DOACs in newly diagnosed AF compared to males [9]. In the ORBIT-AF registry of 10,135 patients at 176 US sites, 42% were female, and females had similar OAC rates to men despite having more functional impairment and lower self-reported quality of life than men [11]. Our study results demonstrated anticoagulation rates were not significantly different by sex, possibly attributable to the effects of an integrated health care system.

### Race/ethnicity

Prior data on racial disparities indicate that Black and Hispanic patients [12, 19] as well as American Indian and Alaskan Native patients may have lower rates of OAC compared to White patients [14]. In addition, a large-scale analysis reported higher rates of stroke and death in Black and Hispanic patients with AF [19, 20]. We found that Black and Asian patients were significantly less likely than White patients to receive guideline-recommended OAC. This is consistent with prior studies showing a disparity in anticoagulation use in underrepresented racial/ethnic groups. There was no significant difference in anticoagulation rates between Hispanic and White patients in our study, although the number of Hispanic patients was relatively low.

When adjusted for CHADS_2_ scores, the observed differences in OAC use only persisted for Black and White patients with lower CHADS_2_ scores of 2 or 3. In contrast, continued disparity in anticoagulation rates was seen between Asian or Pacific Islander and White patients with higher CHADS_2_ scores of 5 or greater. These findings may reflect the role of ethnicity in affecting patient behavior, healthcare literacy, as well as the level of comorbid conditions in these groups, which could drive decisions about anticoagulation, or patient willingness to initiate OAC. Further study is needed to delineate those differences to ensure the equitable access to AF management and treatment across all ethnic groups.

Importantly, our cross-sectional study included significantly higher rates of Black patients (35.5% of total AF patients) than were included in some other registries; the ORBIT I and ORBIT II studies included only 5.0 and 4.9%, respectively [21], and NCDR Pinnacle included 2.9% [22]. This is also true for Asian or Pacific Islander patients of which there were 0.6% in NCDR PINNACLE [22] as compared with 8.3% included in our analysis. Ensuring adequate representation of racial/ethnic groups even in observational studies is critical to better understanding differences in treatment and outcomes between these groups.

### Socioeconomic status

In most studies investigating epidemiological disparities in anticoagulation rates for AF patients, access to care and socioeconomic factors have been proposed barriers to standard care, especially the cost of DOACs [15, 23]. In addition to lower likelihood of treatment with DOACs for AF [15], lower socioeconomic status has been associated with poorer clinical outcomes [23], lower health related quality of life [24], and lower rates of catheter ablation [25]. In contrast, our SES analysis showed no difference in rates of guideline-mandated anticoagulation among all SES quartiles. The SES-SVI groups were well balanced numerically, which suggests that while surprising, this may not be a chance finding. It may be due to the unique model of KP as a simultaneous private insurer and provider, with many patients being on Medicare and eligible for low-cost preferred formulary medications.

### Reasons for lack of anticoagulation

There has been limited contemporary data published on the reasons for lack of anticoagulation in eligible patients with AF. Prior studies have typically surveyed patients and/or prescribers for possible barriers [23, 26, 27], and such barriers include medication cost, access to care, and comorbidities conferring an increased risk of bleeding. The most frequent reason for lack of anticoagulation risk in our AF population was a history of prior or current bleeding or elevated bleeding risk. Patients being lost to follow up and patient refusal were next in frequency, and we noted a high burden of comorbid psychiatric illness. A significant number of patients had one or more barrier to anticoagulation, and importantly, only a small proportion of patients not receiving guideline-recommended OAC were eligible or willing to be anticoagulated. Larger population analyses are needed to characterize these barriers on a broader and more generalizable scale.

### Limitations

Our study has several limitations. We were restricted to using CHADS_2_ score when analyzing our study population given the current limits of the AF Registry; CHADS2VASc or ATRIA would have been better choices given more accurate stroke risk prediction with these scores. Bleeding risk among different subgroups was not assessed in this study, which may be an unmeasured confounding factor. Additionally, our patient experience may not be widely generalizable to all populations and practice settings. The KP model is a closed group-model, integrated healthcare delivery system, with patients receiving care nearly exclusively from Permanente physicians, and with a direct and efficient referral system between primary care and cardiology specialists. Access to care and medication cost is less of an issue for private insurance holders with KP given that that nearly all members have a pharmacy benefit with low drug co-pays. As this was a retrospective cross-sectional study, some recently diagnosed patients with AF may have been counted as not appropriately anticoagulated even though they may not have yet had the opportunity to complete their evaluation for OAC eligibility. Finally, our anticoagulation regimens with dabigatran as the preferred DOAC may not be broadly generalizable to all other U.S. practice settings, where the most common DOAC currently prescribed is apixaban [28].

## Conclusion

In conclusion, among AF patients receiving care within an integrated healthcare system, guideline-recommended anticoagulation rates are reasonably high and do not appear to differ by sex or SES. There were modestly lower rates of anticoagulation among Black and Asian patients compared with White patients. Reasons for lack of anticoagulation were observed to vary significantly, and importantly, only a low proportion of patients who were not anticoagulated were eligible or willing to be anticoagulated. This study has implications for both patients with AF and managing providers with AF, where ensuring maximal benefit from anticoagulation medication must involve understanding the patient and any system-level factors associated with lack of anticoagulation. Further study is needed to delineate methods of intervening to improve appropriate anticoagulation, especially in those patients with AF at the highest thromboembolic risk.

### Supplementary Information

**Additional file 1.**

## Data Availability

The datasets used and/or analyzed during the current study are available from the corresponding author on reasonable request. All data generated or analyzed during this study are included in this published article.
