# Supplementary material for: Gaps in guideline-recommended anticoagulation in patients with atrial fibrillation and elevated thromboembolic risk within an integrated healthcare delivery system
Source: BMC Cardiovasc Disord. 2023 Nov 21;23:578. doi: 10.1186/s12872-023-03607-y (PMC10664365; doi:10.1186/s12872-023-03607-y)
Supplement: Supplementary file 1 — Additional file 1. [file 12872_2023_3607_MOESM1_ESM.pdf]

Supplemental Appendix: Description of Registry Criteria and ICD-10 Codes

Kaiser Permanente Mid Atlantic States (KPMAS) electronic health record system has several registries (Atrial Fibrillation, Hypertension, Congestive Heart Failure, Diabetes Mellitus) which we utilized to base, descriptors of the registry inclusion criteria are outlined below. For the risk factors of coronary artery disease, peripheral arterial disease, and prior stroke, ICD 10 codes were identified and used and are described in further detail below.

<u>Atrial Fibrillation Registry:</u>

This rule evaluates atrial fib or flutter, and if present in Encounter Dx (2 years) or Problem List, is included in the Atrial Fibrillation Registry.

1. Patient is not deceased. AND

2. AGE is >= 18. AND

3. A patient has "Active Coverage" Health Maintenance modifier. AND

4. A patient has a AFIB or AFLUTTER diagnosis code on the (active) Problem List.

OR

1. Patient is not deceased. AND

2. AGE is >= 18. AND

3. A patient has "Active Coverage" Health Maintenance modifier. AND

5. A patient has a AFIB or AFLUTTER diagnosis code on the encounter diagnoses. OR

6. A patient has an EKG impression within that last 90 days that indicates Afib/Aflutter. AND

7. A patient does not have a resolved Afib/Aflutter dx on the Problem List. AND

8. A patient does not have an active dx of Hx of Afib/Aflutter on the Problem List.

<u>Hypertension Registry:</u>

Status: Alive, Active insurance

Diagnoses in Problem List OR Diagnoses in at least 1 visit encounter within the past 1 year OR Diagnoses listed on billing claims

Diagnoses = Hypertension grouper

<u>Congestive Heart Failure Registry:</u>

Status: Alive, Active insurance

Patient enrolled in CHF program (manual process).

Diabetes Mellitus Registry:

This rule evaluates various inclusion criteria and if a patient meets these criteria, he/she is included in the diabetes registry. The rule uses 6 criteria:

1. A patient has living status of alive.

2. A patient has Active Coverage-Reporting Workbench health maintenance modifier.

3. A patient has a diabetes diagnosis from the grouper on the active problem list

OR

4. A patient has at least 2 encounters in a primary care or endocrinology department with a DM diagnoses in the past year.

OR

5. Patient has an active medication from the DM Meds grouper prescribed. AND

6. Patient does not have PCOS or Prediabetes

Coronary Artery Disease:

ICD 10 root groups: I25, I21, T82, I22

Peripheral Artery Disease:

ICD 10 root groups: I73, Z98, Z95

Prior Ischemic Stroke:

ICD 10 root groups: G46, Z86

CHADS2VASC Score:

Congestive Heart Failure (1 point)

Hypertension (1 point)

Age <65 (0 points); Age 65-74 (1 point); Age >75 (2 point);

Diabetes (1 point)

Stroke/TIA/thromboembolism history (2 points)

Vascular Disease (1 point)

Sex Female (1 point); Male (0 points)

CHADS2 Score:

Congestive Heart Failure (1 point)

Hypertension (1 point)

Age >75 (1 point)

Diabetes (1 point)

Stroke/TIA/thromboembolism history (2 points)

ATRIA Score:

History of Stroke (8 points)

Age <65 (0 points); Age 65-74 (3 points); Age 75-84 (5 points); Age ≥85 (6 points)

Female (1 point); Male (0 points)

History of Diabetes (1 point)

History of Congestive Heart Failure (1 point)

History of Hypertension (1 point)

History of Proteinuria (1 point)

eGFR <45 or End-Stage-Renal-Disease (1 point)
